# Efficacy comparison of neoadjuvant chemotherapy, chemoradiotherapy, and chemoimmunotherapy in resectable esophageal squamous cell carcinoma: short- and long-term outcomes from a propensity score-matched analysis

**DOI:** 10.3389/fmed.2026.1994701

**Published:** 2026-10-19

**Authors:** Hai Zhang, Yujing Liang, Chaolin Li, Bomeng Wu, Jiangbo Lin, Wanli Lin

**Affiliations:** 1 Department of Thoracic Surgery, Affiliated Gaozhou People’s Hospital, Guangdong Medical University, Guangdong, China; 2 Department of Thoracic Surgery, Fujian Medical University Union Hospital, Fuzhou, China

**Keywords:** chemoradiotherapy, esophageal squamous cell carcinoma, immunotherapy, neoadjuvant therapy, recurrence

## Abstract

**Introduction:**

This study consecutively enrolled patients with resectable locally advanced esophageal squamous cell carcinoma (ESCC) treated at Fujian Medical University Union Hospital and Gaozhou People's Hospital between January 1, 2,019, and January 1, 2022.

**Methods:**

All patients received neoadjuvant therapy followed by minimally invasive McKeown esophagectomy and were categorized according to treatment modality: neoadjuvant chemotherapy (nCT), chemoradiotherapy (nCRT), or chemoimmunotherapy (nICT). Propensity score matching yielded 78 patients per group. The primary endpoints were 3-year overall survival (OS), recurrence-free survival (RFS), and recurrence patterns.

**Results:**

One-year RFS was significantly lower in the nCT group than in the nCRT and nICT groups (*p* = 0.020), whereas 2-year OS was significantly higher in the nICT group (*p* = 0.018). However, no statistically significant differences were observed in 3-year OS or RFS. Recurrence occurred significantly earlier in the nCT group than in the nICT group (*p* < 0.05), with no statistically significant differences in the other pairwise comparisons.

**Discussion:**

In this retrospective matched-cohort study, nICT did not significantly improve 3-year OS or RFS compared with nCRT, although recurrence appeared to occur later in the nICT group. These findings should be interpreted as hypothesis-generating, as potential long-term immunotherapy effects and radiotherapy-related modulation of long-term benefit could not be assessed. The lower survival observed in the nCT group suggests that chemotherapy alone may be insufficient for ESCC. Prospective trials are warranted to confirm long-term benefits and identify patients most likely to benefit from nICT.

## 1 Introduction

Treatment strategies for resectable locally advanced esophageal squamous cell carcinoma (ESCC) have evolved substantially in recent years. Neoadjuvant chemotherapy (nCT), neoadjuvant immunotherapy (nIT), and neoadjuvant chemoradiotherapy (nCRT) represent three widely adopted treatment paradigms in contemporary clinical practice, each characterized by distinct short-term efficacy profiles and recurrence patterns (1–3). Studies have reported lower pathological complete response (pCR) rates with nCT than with nCRT, although nCT produces significant tumor regression and lymph node clearance. Additionally, in selected patients with locally advanced ESCC or adenocarcinoma, nCT can improve the success rate of surgical resection (2). Research in ESCC has increasingly focused on nIT and nCRT, particularly their short- to mid-term outcomes in resectable locally advanced disease. Comparative analyses suggest similar disease-free survival (DFS) outcomes. Retrospective studies and meta-analyses have reported three-year DFS rates of approximately 60% with both approaches in patients with locally advanced esophageal cancer (3–5). Moreover, no statistically significant differences in DFS curves have been reported over similar follow-up periods (5–7). Five-year overall survival (OS) rates after nCRT combined with surgery range from 40% to 50%. Because nIT was introduced more recently, available OS data are largely limited to three-year follow-up period, with survival rates exceeding 40% (3, 4). However, evidence comparing three-year outcomes and recurrence patterns after nCT, nICT, and nCRT in locally advanced ESCC remains limited. Therefore, this study aimed to investigate differences in three-year outcomes among these three neoadjuvant treatment modalities.

## 2 Materials and methods

### 2.1 Study design and patient population

This dual-center retrospective cohort study consecutively enrolled patients with resectable locally advanced ESCC treated at Fujian Medical University Union Hospital and Gaozhou People's Hospital between January 1, 2019, and January 1, 2022. All patients had pathologically confirmed ESCC and clinical stage cT2-4aN0-3M0 disease, according to the 8th edition of the American Joint Committee on Cancer (AJCC) TNM staging system. The inclusion criteria were (1) age 18–75 years (2), Eastern Cooperative Oncology Group (ECOG) performance status score of 0–1 (3), resectable locally advanced ESCC as assessed by a multidisciplinary team (MDT), and (4) no prior antitumor therapy. The exclusion criteria were (1) distant metastasis (2), prior esophageal surgery or radiotherapy (3), another concurrent malignancy (4), contraindications to immunotherapy (e.g., autoimmune diseases or active infections), and (5) severe cardiac, pulmonary, hepatic, or renal dysfunction.

### 2.2 Neoadjuvant treatment regimens

Patients were assigned to the nCT, nCRT, or nICT group according to the neoadjuvant treatment received. The regimens were as follows:

nCT group: A taxane-based regimen combined with a platinum agent was administered every 3 weeks for 2–3 cycles.

nCRT group: Patients received the same chemotherapy regimen as the nCT group together with intensity-modulated radiation therapy (IMRT). A total dose of 41.4–50.4 Gy was administered in 23–28 fractions once daily, at 1.8–2.0 Gy per fraction, 5 days per week.

nICT group: An immune checkpoint inhibitor (pembrolizumab, tislelizumab, camrelizumab, or sintilimab, each at 200 mg on day 1) was added to the nCT regimen and administered every 3 weeks for 2–3 cycles. Agent selection depended on the patient's financial circumstances and health insurance coverage.

### 2.3 Surgical approach

All patients underwent minimally invasive McKeown esophagectomy, including thoracoscopic esophageal mobilization, laparoscopic gastric mobilization, and cervical anastomosis, 4–8 weeks after completing neoadjuvant treatment. Operations were performed by senior surgeons with extensive experience in minimally invasive esophageal cancer surgery. Standard lymph node dissection encompassed the bilateral recurrent laryngeal nerve chains, paraesophageal, subcarinal, and perigastric lymph nodes, and lymph nodes along the left gastric artery.

### 2.4 Propensity score matching

Propensity score matching (PSM) was performed to reduce selection bias and balance baseline characteristics across the three treatment groups. Propensity scores were estimated using a multinomial logistic regression model that simultaneously included all three treatment groups, with nCT as the reference category. Model covariates included age, sex, body mass index (BMI), hypertension, coronary heart disease, diabetes, tumor location, cT stage, cN stage, and cTNM stage. Patients were matched at a 1:1:1 ratio using nearest-neighbor matching without replacement and a caliper of 0.05 on the propensity score scale. All analyses were performed in R version 4.2 using the “MatchIt” package. This procedure enabled simultaneous matching of the three groups and was reproducible using the specified model, covariates, matching algorithm, caliper, and software. After matching, covariate balance was assessed using standardized mean differences (SMDs), with SMD < 0.10 indicating adequate balance. For covariates with post-matching SMD >0.10 in any pairwise comparison, we further calculated absolute between-group differences (percentage points and number of patients) to facilitate clinical interpretation. The matched cohort comprised 78 patients per group (Table 1), as described in detail in the authors’ previous publication (Zhang et al., 2025, Front. Med. 12:1682377). The main endpoints were 1-year OS, 1-year RFS, and the 2-year analysis. In the matched cohort, the event numbers were 16 for 1-year OS, 39 for 1-year RFS, and 45 for the 2-year analysis. Because these event numbers were limited, adjusting for all five covariates with SMD >0.10 simultaneously in a multivariable Cox model would result in events per variable (EPV) of 3.2, 7.8, and 9.0, respectively, all below the commonly recommended threshold of EPV ≥10. Therefore, to avoid model overfitting and unstable estimates, an additional adjusted sensitivity analysis was not performed. Complete pre- and post-matching SMDs and absolute differences are provided in Table 2.

**Table 1:** Comparison of baseline characteristics among the nCRT, nICT, and nCT groups before and after propensity score matching.

Variable	Before PSM					After PSM				
	nCRT (*n* = 78)	nICT (*n* = 218)	nCT(*n* = 141)	p-value	SMD	nCRT (*n* = 78)	nICT (*n* = 78)	nCT (*n* = 78)	p-value	SMD
Male, n (%)	57 (73.1)	171 (78.4)	109 (77.3)	0.625	0.083	57 (73.1)	63 (80.8)	60 (76.9)	0.522	0.121
Age (years), mean (SD)	61.37 (7.10)	60.52 (6.60)	61.16 (7.38)	0.549	0.081	61.37 (7.10)	62.17 (6.58)	61.35 (7.63)	0.716	0.078
BMI (kg/m^2^), mean (SD)	21.93 (2.73)	22.29 (2.71)	22.23 (2.72)	0.957	0.052	21.93 (2.73)	22.13 (2.35)	22.10 (2.75)	0.873	0.051
Hypertension (+), n (%)	6 (7.7)	36 (16.5)	31 (22.0)	0.025	0.273	6 (7.7)	8 (10.3)	14 (17.9)	0.121	0.206
Coronary heart disease(+), n (%)	1 (1.3)	1 (0.5)	5 (3.5)	0.073	0.152	1 (1.3)	0 (0)	4 (5.1)	0.070	0.235
Diabetes (+), n (%)	4 (5.1)	15 (6.9)	9 (6.4)	0.863	0.049	4 (5.1)	5 (6.4)	5 (6.4)	0.927	0.036
Tumor location, n (%)				0.008	0.266				0.085	0.078
Upper	7 (9.0)	18 (8.3)	12 (8.5)			7 (9.0)	7 (9.0)	8 (10.3)		
Middle	56 (71.8)	111 (50.9)	70 (49.6)			56 (71.8)	41 (52.6)	43 (55.1)		
Lower	15 (19.2)	89 (40.8)	59 (41.8)			15 (19.2)	30 (38.5)	27 (34.6)		
cTNM, n (%)				0.967	0.052				0.558	0.163
II	28 (35.9)	80 (36.7)	56 (39.7)			28 (35.9)	19 (24.4)	27 (34.6)		
III	45 (57.7)	123 (56.4)	77 (54.6)			45 (57.7)	52 (66.7)	45 (57.7)		
IV	5 (6.4)	15 (6.9)	8 (5.7)			5 (6.4)	7 (9.0)	6 (7.7)		
cT, n (%)				0.377	0.176				0.719	0.100
2	9 (11.5)	45 (20.6)	32 (22.7)			9 (11.5)	12 (15.4)	15 (19.2)		
3	66 (84.6)	165 (75.7)	104 (73.8)			66 (84.6)	63 (80.8)	59 (75.6)		
4	3 (3.8)	8 (3.7)	5 (3.5)			3 (3.8)	3 (3.8)	4 (5.1)		
cN, n (%)				0.725	0.106				0.653	0.199
0	22 (28.2)	42 (19.3)	30 (21.3)			22 (28.2)	13 (16.7)	16 (20.5)		
1	45 (57.7)	145 (66.5)	90 (63.8)			45 (57.7)	49 (62.8)	47 (60.3)		
2	9 (11.5)	21 (9.6)	15 (10.6)			9 (11.5)	11 (14.1)	11 (14.1)		
3	2 (2.6)	10 (4.6)	6 (4.3)			2 (2.6)	5 (6.4)	4 (5.1)		

**Table 2:** Post-matching balance for covariates with SMD >0.10 in at least one pairwise comparison.

Covariate	Max pre-matching SMD	Max post-matching SMD	Max absolute difference after matching
Male, n (%)	0.083	0.121	nCRT vs. nICT: 57/78 (73.1%) vs. 63/78 (80.8%), difference 7.7 percentage points (6 patients)
Hypertension (+), n (%)	0.273	0.206	nCRT vs. nCT: 6/78 (7.7%) vs. 14/78 (17.9%), difference 10.3 percentage points (8 patients)
Coronary heart disease (+), n (%)	0.152	0.235	nICT vs. nCT: 0/78 (0%) vs. 4/78 (5.1%), difference 5.1 percentage points (4 patients)
cTNM, n (%)	0.052	0.163	nCRT vs. nICT: stage II 28/78 (35.9%) vs. 19/78 (24.4%), difference 11.5 percentage points (9 patients)
cN, n (%)	0.106	0.199	nCRT vs. nICT: cN0 22/78 (28.2%) vs. 13/78 (16.7%), difference 11.5 percentage points (9 patients)

### 2.5 Study endpoints and definitions

The primary endpoint was the 3-year overall survival (OS) rate, defined as the time from surgery to death from any cause or the last follow-up date. Secondary endpoints included recurrence-free survival (RFS) and recurrence patterns. RFS was defined as the time from surgery to the first recurrence (locoregional recurrence or distant metastasis) or death from any cause. Recurrence was classified by its anatomical relationship to the surgical field, rather than the radiotherapy field. In-field recurrence was defined as recurrence within the original resection field or an adjacent anatomical region, including the anastomotic site, gastric conduit, primary tumor bed, and regional lymph nodes. Out-of-field recurrence was defined as recurrence outside the surgical field, including non-regional lymph nodes and distant organs. Because only patients in the nCRT group received radiotherapy, “in-field” and “out-of-field” refer here to the surgical or anatomical field and should not be confused with the radiation field. These categories broadly correspond to locoregional and distant recurrence, respectively.

### 2.6 Follow-up protocol

Patients were followed up every 3 months during the first 2 years after surgery and every 6 months from the third year onward. Assessments included physical examination, hematological tests (SCC-Ag and CEA tumor markers), contrast-enhanced chest CT, cervical and abdominal ultrasound/contrast-enhanced CT, and upper gastrointestinal endoscopy. Suspected recurrence or metastasis was evaluated using PET-CT or MRI of the relevant site. The last follow-up date was December 31, 2024.

### 2.7 Statistical analysis

OS and RFS at 1, 2, and 3 years were estimated using the Kaplan–Meier method. Survival curves were plotted using GraphPad Prism 9 (GraphPad Software, La Jolla, CA, USA). A *P* < 0.05 was considered statistically significant.

## 3 Results

After three-group propensity score matching (PSM), each group included 78 patients (total *N* = 234). Five covariates had post-matching SMD values exceeding 0.10 in at least one pairwise comparison:

Male (max SMD=0.121; nCRT vs. nICT: 57/78 [73.1%] vs. 63/78 [80.8%], absolute difference 7.7 percentage points, 6 patients)

Hypertension (max SMD=0.206; nCRT vs. nCT: 6/78 [7.7%] vs. 14/78 [17.9%], absolute difference 10.3 percentage points, 8 patients)

Coronary heart disease (max SMD=0.235; nICT vs. nCT: 0/78 [0%] vs. 4/78 [5.1%], absolute difference 5.1 percentage points, 4 patients)

cTNM (max SMD=0.163; nCRT vs. nICT: stage II: 28/78 [35.9%] vs. 19/78 [24.4%], absolute difference 11.5 percentage points, 9 patients)

cN (max SMD=0.199; nCRT vs. nICT: cN0: 22/78 [28.2%] vs. 13/78 [16.7%], absolute difference 11.5 percentage points, 9 patients)

The maximum absolute difference was nine patients, while the remaining absolute differences ranged from four to eight patients. The complete pre- and post-matching SMDs and absolute differences are provided in Table 2.

### 3.1 One-year OS and RFS

The one-year OS rates were 91.03%, 94.87%, and 93.59% in the nCRT, nICT, and nCT groups, respectively. No statistically significant difference was observed in the one-year OS among the three groups (*p* = 0.615), as shown in Figure 1.

**Figure 1:**
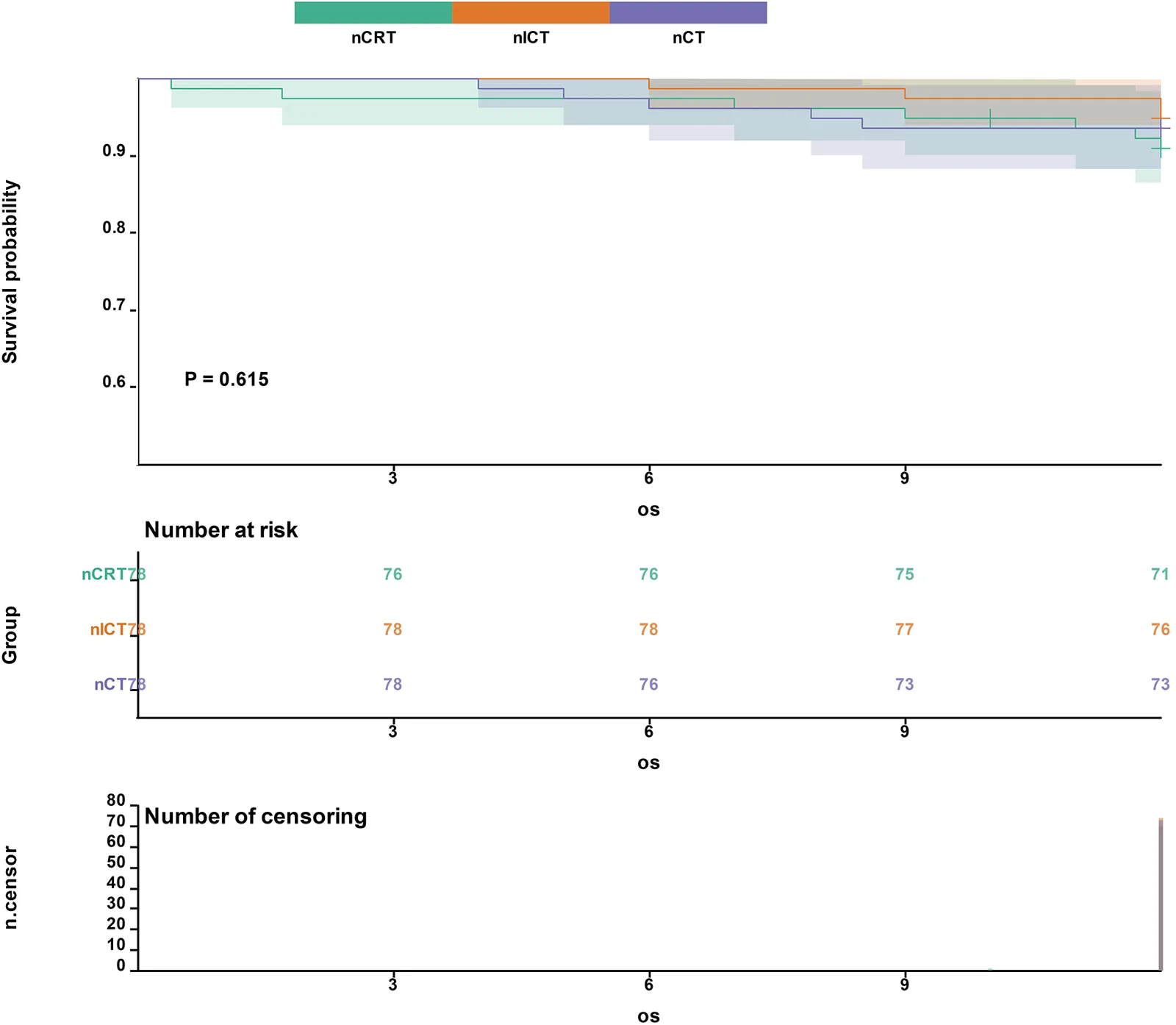
Comparison of one-year overall survival (OS) among the nCT, nICT, and nCRT groups. The one-year OS rates were 91.03% in the nCRT group, 94.87% in the nICT group, and 93.59% in the nCT group, with no statistically significant difference observed (*p* = 0.615).

The one-year RFS rates were 82.05%, 92.31%, and 75.64% in the nCRT, nICT, and nCT groups, respectively. The overall difference among groups was statistically significant (*p* = 0.020)(Figure 2).

**Figure 2:**
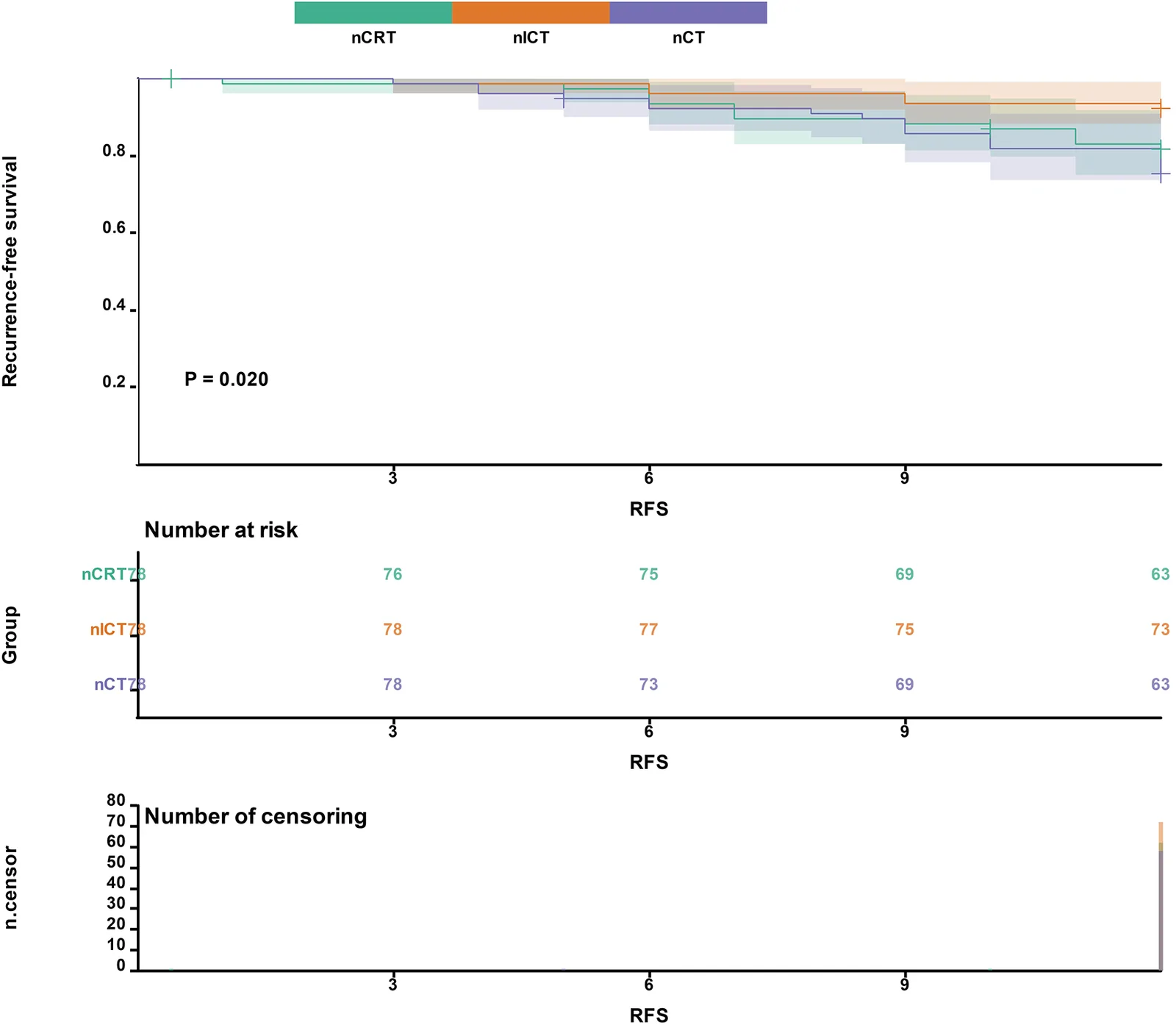
Comparison of one-year recurrence-free survival (RFS) among the nCT, nICT, and nCRT groups. The one-year RFS rate was significantly higher in the nICT group (92.31%) compared with the nCRT (82.05%) and nCT (75.64%) groups (*p* = 0.020).

### 3.2 Two-year OS and RFS

The two-year OS was significantly higher in the nICT group (91.03%) than in the nCRT (78.21%) and nCT (73.08%) groups (*p* = 0.018)(Figure 3).

**Figure 3:**
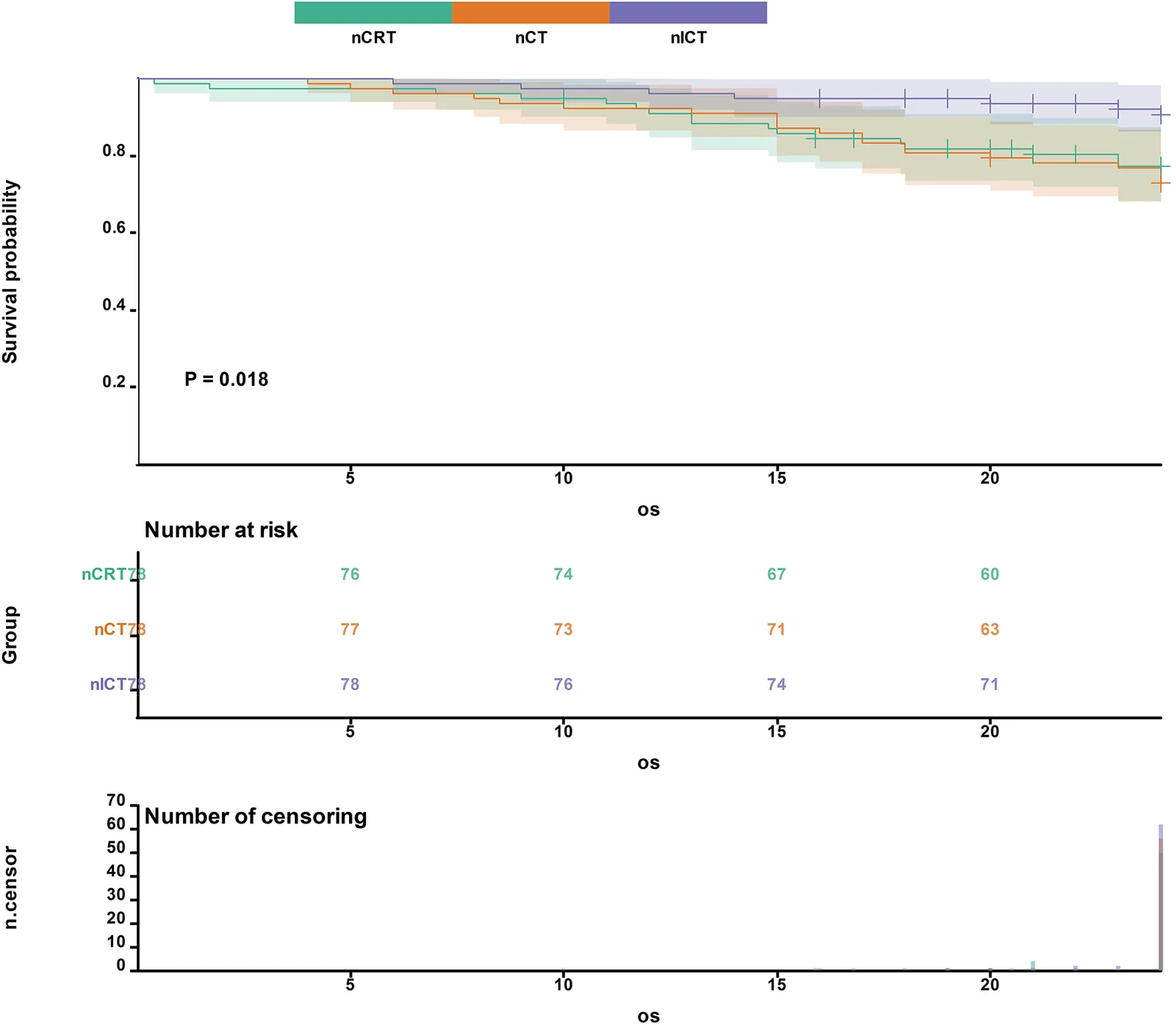
Comparison of two-year overall survival (OS) among the nCT, nICT, and nCRT groups. The nICT group demonstrated a significantly higher OS rate (91.03%) compared with the nCRT (78.21%) and nCT (73.08%) groups (*p* = 0.018).

Two-year RFS was highest in the nICT group (79.49%), followed by the nCRT (76.92%) and nCT (75.64%) groups. However, the differences were not statistically significant (*p* = 0.349)(Figure 4).

**Figure 4:**
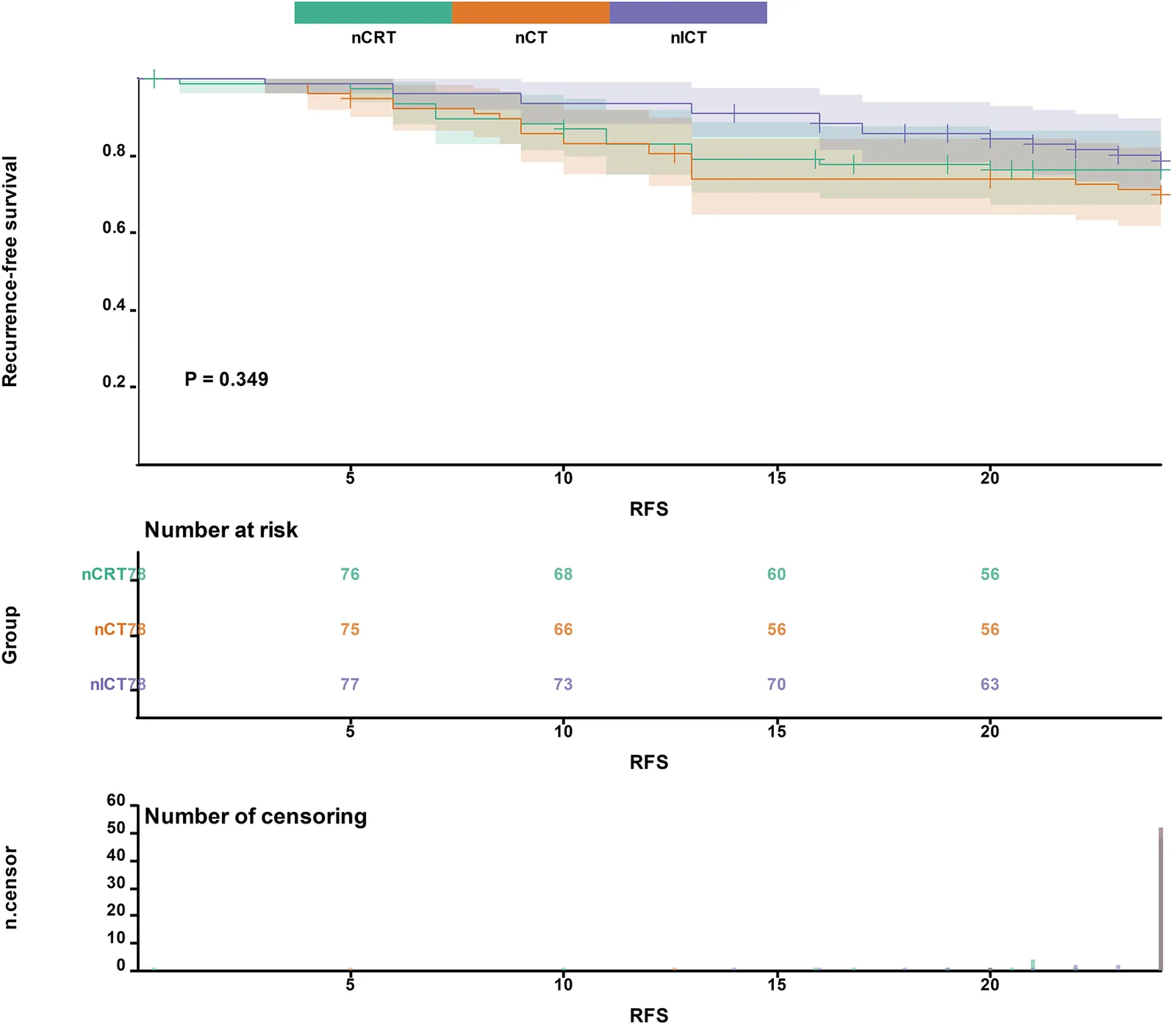
Comparison of two-year recurrence-free survival (RFS) among the nCT, nICT, and nCRT groups. Although the nICT group showed the highest RFS rate (79.49%), followed by the nCRT (76.92%) and nCT (75.64%) groups, the differences were not statistically significant (*p* = 0.349).

### 3.3 Three-year OS and RFS

Although the three-year OS rate in the nICT group (82.05%) was higher than that in the nCRT (74.36%) and nCT (65.38%) groups, the differences were not statistically significant (*p* = 0.110)(Figure 5).

**Figure 5:**
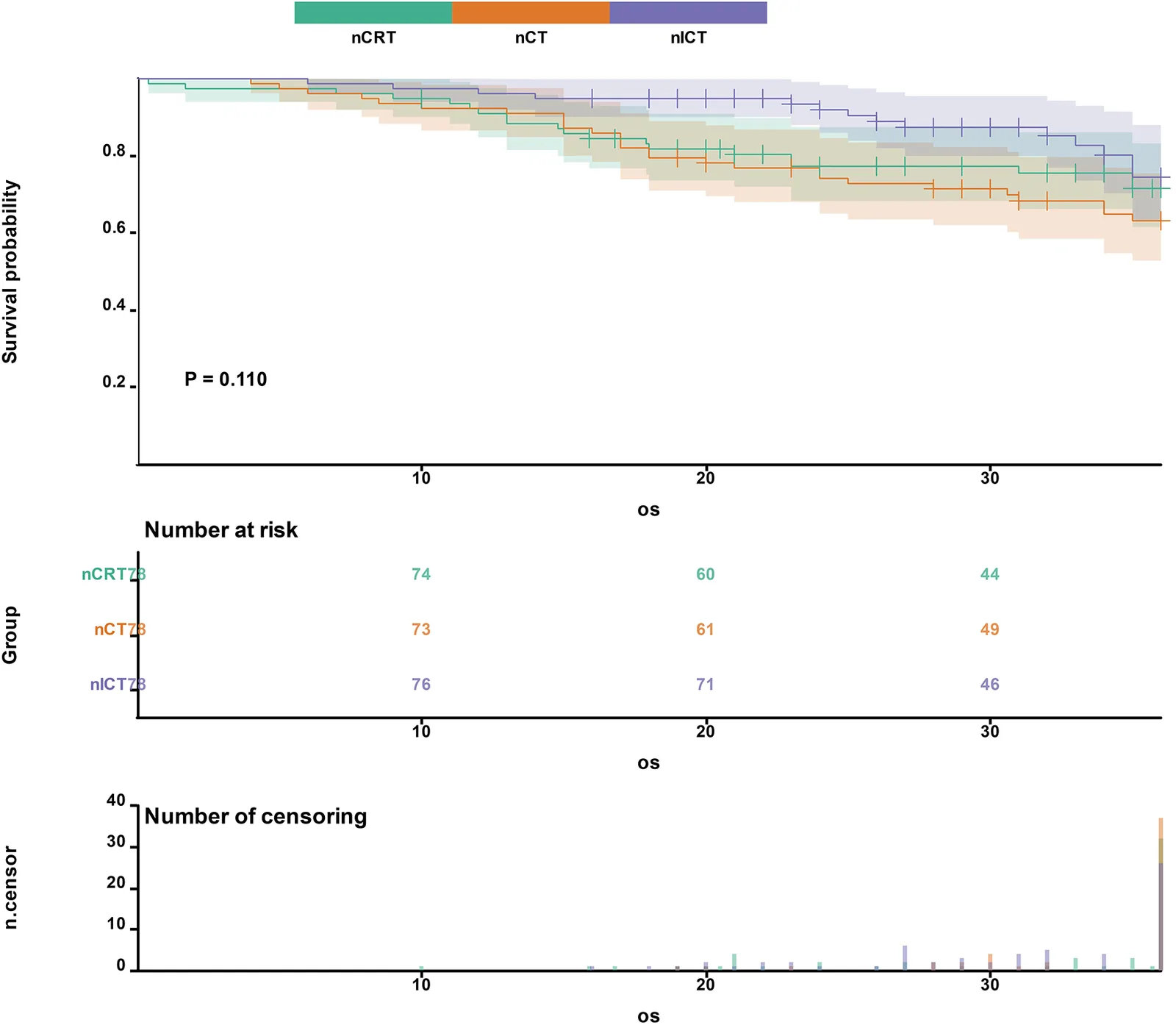
Comparison of three-year overall survival (OS) among the nCT, nICT, and nCRT groups. Although the nICT group exhibited the highest OS rate (82.05%), followed by the nCRT (74.36%) and nCT (65.38%) groups, the differences were not statistically significant (*p* = 0.110).

The three-year RFS rates were 74.36%, 71.79%, and 67.95% in the nICT, nCRT, and nCT groups, respectively. No statistically significant difference was detected among the groups (*p* = 0.564)(Figure 6).

**Figure 6:**
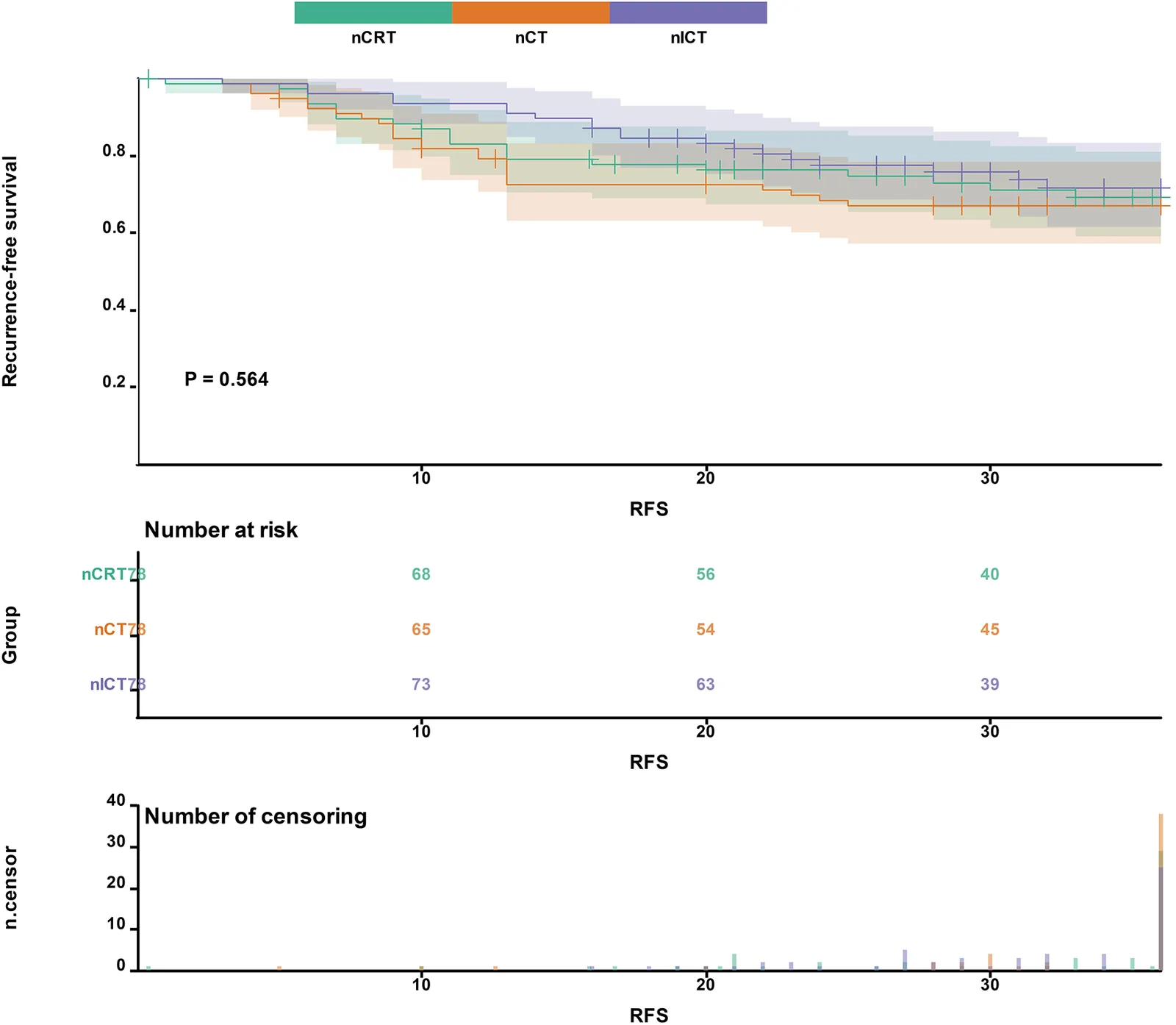
Comparison of three-year recurrence-free survival (RFS) among the nCT, nICT, and nCRT groups. The RFS rates were 74.36% in the nICT group, 71.79% in the nCRT group, and 67.95% in the nCT group, with no statistically significant differences detected (*p* = 0.564).

### 3.4 Patterns of recurrence

Postoperative recurrence was classified anatomically as in-field or out-of-field recurrence.

In-field recurrence comprised tumor relapse within the original surgical resection field or an adjacent anatomical region, including the primary tumor site, surgical margin, adjacent tissues, and regional lymph nodes of the esophageal tumor area.

Out-of-field recurrence comprised tumor relapse outside the surgical field, including regional lymphatic drainage areas or adjacent anatomical structures without direct anatomical continuity with the surgical field, as well as non-regional lymph nodes and distant organs.

The earliest postoperative recurrence occurred at 3 months in the nICT group, 1 month in the nCRT group, and 3 months in the nCT group. Median times to recurrence were 16 (IQR: 10–22), 11 (IQR: 7–17), and 10 (IQR: 6–13) months, respectively. Time to recurrence differed significantly between the nICT and nCT groups (*p* = 0.014). Conversely, differences between the nICT and nCRT groups (*p* = 0.14) and between the nCT and nCRT groups (*p* > 0.05) were not statistically significant (Figure 7A).

**Figure 7:**
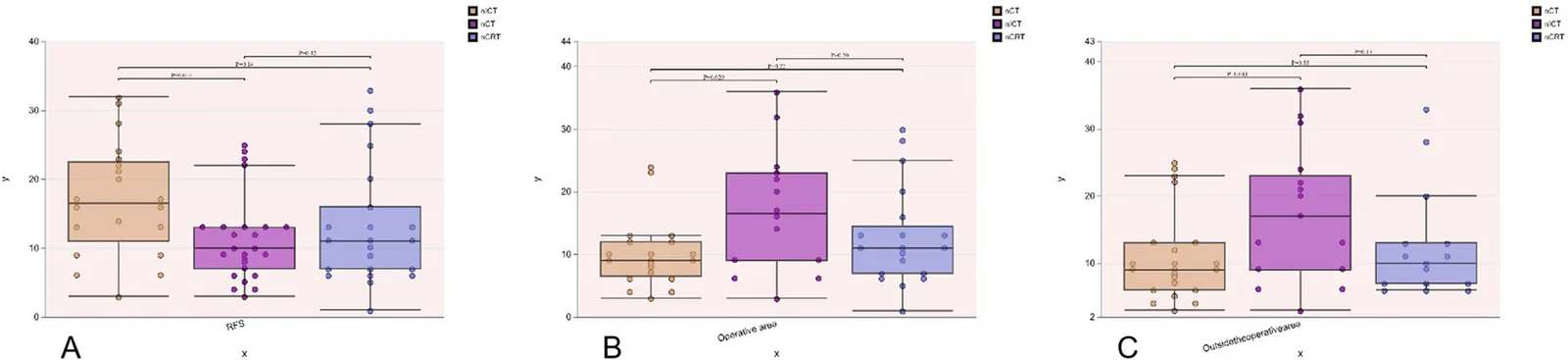
**(A)**:comparison of time to recurrence among the nCT, nICT, and nCRT groups. The nICT group demonstrated a significantly prolonged time to recurrence compared with the nCT group (median 16 vs. 10 months; *p* = 0.014). No statistically significant differences were observed between the nICT and nCRT groups (*p* = 0.14) or between the nCT and nCRT groups (*p* > 0.05). **(B)**: Comparison of time to in-field recurrence among the nCT, nICT, and nCRT groups. The nICT group exhibited a significantly prolonged time to in-field recurrence compared with the nCT group (median 16 vs. 9 months; *p* = 0.029). No statistically significant differences were observed between the nCT and nCRT groups or between the nICT and nCRT groups (*p* > 0.05). **(C)**: Comparison of time to out-of-field recurrence among the nCT, nICT, and nCRT groups. The nICT group demonstrated a significantly delayed time to out-of-field recurrence compared with the nCT group (median 17 vs. 9 months; *p* = 0.041). No statistically significant differences were observed between the nICT and nCRT groups or between the nCT and nCRT groups (*p* > 0.05).

In-field recurrence occurred in 20 patients in the nCT group, 14 in the nICT group, and 18 in the nCRT group. The earliest in-field recurrence occurred 3 months postoperatively in both the nCT and nICT groups, and 1 month after surgery in the nCRT group. Median times to in-field recurrence were 9 (IQR: 6–12), 16 (IQR: 8–23), and 11 (IQR: 7–17) months, respectively. Time to in-field recurrence was significantly longer in the nICT group than in the nCT group (*p* = 0.029). Neither the nCT–nCRT nor the nICT–nCRT comparison was statistically significant (*p* > 0.05, Figure 7B).

Out-of-field recurrence occurred in 22 patients in the nCT group, 14 in the nICT group, and 15 in the nCRT group. The earliest out-of-field recurrence occurred 3 months postoperatively in both the nCT and nICT groups and 6 months after surgery in the nCRT group. Median times to out-of-field recurrence were 9 (IQR: 6–13), 17 (IQR: 9–24), and 10 (IQR: 7–13) months, respectively. Time to out-of-field recurrence was significantly longer in the nICT group than in the nCT group (*p* = 0.041). Neither the nICT–nCRT nor the nCT–nCRT comparison was statistically significant (*p* > 0.05, Figure 7C).

## 4 Discussion

Neoadjuvant immune checkpoint inhibition combined with chemotherapy has attracted increasing interest in locally advanced ESCC. Compared with nCT, nICT has achieved a higher pCR rate comparable to that of nCRT, without significantly increasing treatment-related adverse events during neoadjuvant treatment or the perioperative period. Advantages have been observed for some parameters. Comparisons of mid- to long-term survival after nICT, nCT, and nCRT remain a major focus of research in locally advanced ESCC (8–10).

This study compared three-year OS and RFS after nICT, nCT, and nCRT. One-year OS did not differ significantly among groups (*p* = 0.615). One-year RFS was numerically higher in the nICT group (92.31%) than in the nCRT (82.05%) and nCT (75.64%) groups, with an overall p value of 0.020. Two-year OS was significantly higher in the nICT group (91.03%) than in the nCRT (78.21%) and nCT (73.08%) groups (*p* = 0.018). At three years, the nICT group retained a numerical advantage in OS (82.05%) and RFS (74.36%), although the differences were not statistically significant (*p* = 0.110 and 0.564, respectively), which may be attributable to the relatively short follow-up duration or small sample size.

Mechanistically, nICT may delay tumor recurrence or metastasis through the “long-tail” effect of immunotherapy, ultimately contributing to the substantially higher two-year OS rate. However, the absence of a significant RFS improvement over nCRT suggests that improved locoregional control with radiotherapy may offset the long-term survival benefit of immunotherapy. The lower survival observed in the nCT group (three-year OS = 65.38%) further underscores the limited efficacy of chemotherapy alone.

The neoadjuvant strategy that provides the greatest OS and event-free survival (EFS) benefit remains uncertain. An interim analysis of a phase III randomized trial reported a one-year EFS of 77.9% with neoadjuvant toripalimab plus chemotherapy and 64.3% with chemotherapy alone (HR = 0.62, 95% CI: 0.39–1.00, *p* = 0.05). The one-year OS was 94.1% and 83.0%, respectively (HR = 0.48, 95% CI: 0.24–0.97, *p* = 0.037), supporting a short-term survival benefit with nICT over nCT (11). In contrast, a recent meta-analysis reported numerically improved PFS with nICT compared with nCT, although the difference was not statistically significant (HR: 0.50, *p* = 0.072) (12). In a propensity score-matched comparison of nICT and nCRT, Zhao et al. (13) reported higher one-year DFS with nCRT (94.3% vs. 81.8%), with a similar trend at two years. The KEYSTONE-002 phase III trial comparing nIT and nCRT in locally advanced ESCC is expected to provide further evidence on the optimal neoadjuvant strategy (14).

For locally advanced esophageal malignancies, neoadjuvant therapy combined with surgery improves survival compared with surgery alone, although recurrence rates exceed 40% (15, 16). Postoperative recurrence compromises the long-term survival of patients with esophageal cancer. Previous research has demonstrated that neoadjuvant therapy primarily improves locoregional control without substantially reducing systemic recurrence. Understanding postoperative recurrence patterns is therefore essential for optimizing long-term cancer management and improving survival outcomes. Tumor recurrence is generally categorized as local recurrence, regional lymph node metastasis, or distant metastasis. Local recurrence typically involves the primary tumor site or anastomotic region and is common after non-R0 resection or a poor response to neoadjuvant therapy. Lymphatic spread is a major metastatic route in esophageal cancer, and nodal recurrence can occur despite neoadjuvant treatment (17). Postoperative pathological lymph node stage (ypN stage) has been identified as an important predictor of recurrence following neoadjuvant therapy (18). In this study, nICT showed advantages in delaying postoperative recurrence and reducing both in-field and out-of-field recurrence risks. Specifically, the median overall time to recurrence was significantly longer with nICT (16 months) than with nCT (10 months, *p* = 0.014), whereas the difference from nCRT (11 months, *p* = 0.14) was not statistically significant. This finding suggests that sustained immunologic effects of nICT may suppress minimal residual disease and thereby delay recurrence. In contrast, the shorter recurrence interval with nCT suggests relatively weaker locoregional and systemic control with chemotherapy alone. The intermediate median recurrence time with nCRT may reflect improved local control with radiotherapy, which partially offsets the long-term benefit of immunotherapy.

For in-field recurrence, the median time to recurrence was significantly longer with nICT (16 months) than with nCT (9 months, *p* = 0.029), whereas the difference from nCRT (11 months) was not substantial. The 2-month longer median interval with nCRT than with nCT may reflect improved local control with radiotherapy. In contrast, nICT may prolong the time to local recurrence by activating antitumor immune responses in the tumor microenvironment. The absence of a significant difference in median time to recurrence between nICT and nCRT (*p* > 0.05) suggests that immune activation and radiation-induced cytotoxicity may provide complementary mechanisms of local control, warranting validation in larger cohorts.

The median time to out-of-field recurrence was significantly longer with nICT (17 months) than with nCT (9 months, *p* = 0.041), whereas the difference from nCRT (10 months) was not statistically significant. These findings further support the hypothesis that immunotherapy may delay distant relapse by eliciting systemic antitumor immune responses that suppress micrometastatic disease. Notably, the median interval with nCRT was shorter than with nICT and longer than with nCT (9 months), suggesting that chemoradiotherapy may partially control micrometastatic foci, albeit less effectively than systemic immune modulation with immunotherapy. Overall, nICT was associated with a longer time to recurrence than nCRT, without statistical significance, even though 3-year OS and RFS did not differ significantly. This pattern may be consistent with a context-dependent interaction between immunotherapy and radiotherapy. However, our retrospective clinical data cannot establish whether radiotherapy enhanced, offset, or had no effect on the long-term benefit of immunotherapy. This interpretation should therefore be regarded as hypothesis-generating rather than mechanistic. Ma and Baran (19) discussed the rationale for integrating immunotherapy and radiotherapy in ESCC, highlighting their potentially complementary effects and the need for prospective validation and biomarker-based patient selection before broad adoption. Given the limited literature on recurrence patterns after nICT, nCRT, and nCT, large prospective clinical trials are warranted.

Recurrence and minimal residual disease (MRD) remain key determinants of treatment failure after ESCC resection. Perioperative circulating tumor DNA (ctDNA) has attracted increasing interest for recurrence-risk assessment and biology-driven surveillance. Klacking and Ma (20) discussed its potential to detect MRD and guide postoperative surveillance in ESCC. Unlike conventional imaging, ctDNA analysis can identify molecular recurrence months before radiographic progression, potentially allowing earlier intervention. In this study, recurrence occurred numerically later in the nICT group, although the 3-year OS and RFS did not differ significantly. This observation could reflect a sustained immunologic effect that attenuates MRD, chance, residual confounding, or differences in surveillance. Because we did not measure perioperative ctDNA, MRD markers, or longitudinal immune responses, we cannot determine whether nICT altered molecular recurrence dynamics. Future studies integrating perioperative ctDNA and other MRD-based approaches are warranted to clarify whether nICT delays recurrence by eradicating MRD and to identify patients most likely to benefit.

Research on terminally exhausted CD8+ T-cell states provides a biological framework for understanding the heterogeneity and durability of checkpoint-blockade responses (21). These states may contribute to differences in treatment response and relapse timing after immunotherapy. However, longitudinal immune responses, T-cell exhaustion states, MRD, and relevant biomarkers were not measured in this cohort. This literature therefore contextualizes our observations rather than demonstrating mechanisms operating in the study population. Prospective trials incorporating longitudinal immune monitoring and biomarker-based stratification are warranted to validate the long-term benefits of nICT and identify patients most likely to benefit from this treatment approach.

This study has several limitations. First, its retrospective dual-center design implies that residual confounding cannot be entirely excluded despite PSM. Despite three-group PSM, five covariates still had SMD >0.10 in at least one pairwise comparison after matching, suggesting possible residual confounding. Because the matched cohort included only 78 patients per group and the number of events was limited (16 for 1-year OS, 39 for 1-year RFS, and 45 for the 2-year analysis), an additional multivariable-adjusted sensitivity analysis was not performed to avoid overfitting and unstable estimates. Therefore, the findings should be interpreted as associations rather than causal effects. Larger studies with more events are needed to confirm these results.Second, the matched cohort was relatively small (*n* = 78 per arm), limiting statistical power and precision. Third, the immunotherapy agents were heterogeneous, comprising pembrolizumab, tislelizumab, camrelizumab, and sintilimab. Agent selection was largely influenced by financial circumstances and insurance coverage rather than random assignment, potentially introducing selection bias and confounding treatment comparisons. Fourth, the absence of PD-L1 expression and tumor mutational burden data precluded assessment of these important predictive biomarkers. Fifth, follow-up was limited to 3 years, preventing assessment of longer-term survival and late toxicities. Sixth, radiotherapy doses were not standardized and ranged from 41.4 to 50.4 Gy, potentially affecting efficacy and toxicity. Seventh, the time-to-recurrence analyses in Figures 7A–C included only patients who experienced recurrence, excluding those who remained recurrence-free or died before recurrence. These conditional analyses may introduce selection bias and do not account for death as a competing event. Therefore, they do not estimate cumulative recurrence incidence in the full matched cohort and should be interpreted as exploratory and descriptive only. Future time-to-event or competing-risk analyses of the entire matched population are warranted. Collectively, these limitations may introduce residual confounding and limit the generalizability of our findings. Accordingly, the present results should be viewed as hypothesis-generating and warrant confirmation in prospective randomized trials.

In conclusion, nICT was not associated with a statistically significant improvement in 3-year OS or RFS compared with nCRT in this retrospective matched cohort, although recurrence occurred later descriptively in the nICT group. Whether this observation reflects a “long-tail” immunotherapy effect, suppression of minimal residual disease, or an influence of radiotherapy on long-term outcomes remains hypothetical. These mechanisms could not be assessed because longitudinal immune responses, minimal residual disease, and relevant biomarkers were not measured. The early differences may reflect chance, residual confounding, or variation in surveillance practices. The lower survival observed in the nCT group suggests that chemotherapy alone may be insufficient for ESCC, although this finding requires further validation. The optimal neoadjuvant strategy should be individualized according to patient and disease characteristics. Given the retrospective nature of this study, large-scale prospective trials are warranted to validate the long-term benefits of nICT and identify patients most likely to benefit.

## Data Availability

The raw data supporting the conclusions of this article will be made available by the authors, without undue reservation.
